# Selection of Reference Genes for the Normalization of RT-qPCR Data in Gene Expression Studies in Insects: A Systematic Review

**DOI:** 10.3389/fphys.2018.01560

**Published:** 2018-11-06

**Authors:** Jing Lü, Chunxiao Yang, Youjun Zhang, Huipeng Pan

**Affiliations:** ^1^Key Laboratory of Bio-Pesticide Innovation and Application of Guangdong Province, Department of Entomology, South China Agricultural University, Guangzhou, China; ^2^State Key Laboratory for Conservation and Utilization of Subtropical Agro-bioresources, South China Agricultural University, Guangzhou, China; ^3^Department of Plant Protection, Institute of Vegetables and Flowers, Chinese Academy of Agricultural Sciences, Beijing, China

**Keywords:** RT-qPCR, reference genes, SYBR green method, experimental factors, analysis tools

## Abstract

Reverse transcriptase-quantitative polymerase chain reaction (RT-qPCR) is a reliable technique for quantifying expression levels of targeted genes during various biological processes in numerous areas of clinical and biological research. Selection of appropriate reference genes for RT-qPCR normalization is an elementary prerequisite for reliable measurements of gene expression levels. Here, by analyzing datasets published between 2008 and 2017, we summarized the current trends in reference gene selection for insect gene expression studies that employed the most widely used SYBR Green method for RT-qPCR normalization. We curated 90 representative papers, mainly published in 2013–2017, in which a total of 78 insect species were investigated in 100 experiments. Furthermore, top five journals, top 10 frequently used reference genes, and top 10 experimental factors have been determined. The relationships between the numbers of the reference genes, experimental factors, analysis tools on the one hand and publication date (year) on the other hand was investigated by linear regression. We found that the more recently the paper was published, the more experimental factors it tended to explore, and more analysis tools it used. However, linear regression analysis did not reveal a significant correlation between the number of reference genes and the study publication date. Taken together, this meta-analysis will be of great help to researchers that plan gene expression studies in insects, especially the non-model ones, as it provides a summary of appropriate reference genes for expression studies, considers the optimal number of reference genes, and reviews the average number of experimental factors and analysis tools per study.

## Introduction

Reverse transcriptase-quantitative polymerase chain reaction (RT-qPCR) is a premier molecular biology tool and a powerful method for quantification of gene expression levels in real-time (Vandesompele et al., 2002). Although RT-qPCR is one of the most efficient, reliable, and reproducible techniques to quantify gene expression, multiple factors, including the quality and integrity of RNA samples, efficiency of cDNA synthesis, and PCR efficiency, can significantly influence signal normalization (Bustin et al., 2005; Strube et al., 2008). RT-qPCR generally involves normalization of expression levels of multiple genes to the expression levels of a suite of stable reference genes. Even though reference gene transcript levels should ideally be stable across a range of different conditions, previous studies have shown that expression of many commonly used reference genes differs dramatically under different treatment conditions (Kalushkov and Hodek, 2004; Bustin et al., 2013). It is clear that the expression level of many reference genes is condition-specific and accordingly, there is no universal gene that can be used for internal control for all application scenarios, strongly indicating the necessity of conducting custom reference gene selection for RT-qPCR analyses on a case-by-case basis, even for the same species.

Over the last 10 years, RT-qPCR has been increasingly used in genome/transcriptome expression studies in insect species. Furthermore, considerable advancements have been made for identification and validation of appropriate reference genes across various biotic and abiotic experimental conditions in many insect species (Table 1). In RT-qPCR experiments, SYBR Green and TaqMan probes have been the two most frequently used methodologies, with the SYBR Green method being utilized much more frequently. Here, we have summarized only the studies that used the SYBR Green method. It is well known that characterization of reference genes is an onerous task requiring well-designed molecular experiments followed by elaborate computational analyses (Andersen et al., 2004; Pfaffl et al., 2004). Therefore, a comprehensive summary of published sets of experimentally validated reference genes in conjunction with the description of relevant experimental conditions and analysis tools would be timely (Sang et al., 2017).

**Table 1:** Summary of the reference gene studies in insects from 2008 to 2017.

**Insect species**	Reference genes^*^	**Experimental conditions**	**Analysis tools**	**References**
**COLEOPTERA**				
*Leptinotarsa decemlineata*	*Actin1, Actin2, ARF1, ARF4, TATA1, TATA2, RPL4, RPL8, EF1A*	Developmental stage, tissue, insecticide	*geNorm, Normfinder, BestKeeper*	Shi et al., 2013
*Diabrotica virgifera virgifera*	*Actin, EF1A, RPS9, GAPDH, β-tubulin*	Developmental stage, tissue, dsRNA exposure, Bt toxin exposure	*geNorm, Normfinder, BestKeeper, ΔC_*t*_ method*	Rodrigues et al., 2013
*Hippodamia convergens*	*28S, 18S, Actin, EF1A, GAPDH, CypA, V-ATPase A*	Developmental stage, tissue, sex, temperature, photoperiod, dsRNA exposure	*geNorm, Normfinder, BestKeeper, ΔC_*t*_ method, RefFinder*	Pan et al., 2015b
*Coccinella septempunctata*	*28S, 18S, 16S, NADH, EF1A, Actin, α-tubulin, ArgK*	Developmental stage, tissue, dsRNA exposure	*geNorm, Normfinder, BestKeeper, ΔC_*t*_ method, RefFinder*	Yang et al., 2016
*Coleomegilla maculata*	*28S, 18S, 16S, 12S, Actin, EF1A, GAPDH, ArgK, V-ATPase A, RPS24, HSP70, HSP90, a-tubulin, NADH, RPS18, RPL4*	Developmental stage, tissue, dsRNA exposure	*geNorm, Normfinder, BestKeeper, ΔC_*t*_ method, RefFinder*	Yang et al., 2015c
*Tribolium castaneum*	*Actin, RPS3, RPS6, RPS18, RPS13, E-cadherin, Syntaxin1, Syntaxin6*	Fungal infection	*geNorm, Normfinder*	Lord et al., 2010
	*Actin, GAPDH, RPL13, RPS3, RPS6, RPS18, E-cadherin, Syntaxin1, Syntaxin6*	Developmental stage, tissue	*geNorm, Normfinder*	Toutges et al., 2010
	*Actin, β-tubulin, GAPDH, RPS3, RPL13, RPS18, E-cadherin*	Developmental stage, UV irradiation	*geNorm, Normfinder, BestKeeper*	Sang et al., 2015
*Galeruca daurica*	*Actin, GAPDH, GST, RPL32, SDHA, TATA, α-tubulin, β-tubulin, HSP70, CYP6*	Developmental stage, tissue, sex, temperature, diapause, and non-diapause adults	*geNorm, Normfinder, BestKeeper, ΔC_*t*_ method*	Tan et al., 2017
*Agrilus planipennis*	*Actin, β-tubulin, GAPDH, RPL7, EF1A, UBQ*	Developmental stage, tissue	*geNorm, Normfinder, BestKeeper*,	Rajarapu et al., 2012
*Mylabris cichorii*	*RPL22, RPL13, RPS27, Actin, β-tubulin, UBC, UBE2C, UBE3A, EF1A, TATA*	Sex	*geNorm, Normfinder*	Wang Y. et al., 2014
*Colaphellus bowringi*	*GAPDH, RPL32, RPL19, EF1A, TATA, TATA1, Actin1, Actin2, α-tubulin, α-tubulin 1, β-tubulin*	Developmental stage, sex, population, photoperiod	*geNorm, Normfinder, BestKeeper, RefFinder*	Tan et al., 2015
*Cryptolestes ferrugineus*	*SDHA, Cyclin A, γ-tubulin, α-tubulin, EF1A, GAPDH, RPL13, RPS13, Actin*	Developmental stage, population	*geNorm, Normfinder, BestKeeper, ΔC_*t*_ method*	Tang et al., 2017
*Anoplophora glabripennis*	*SDFS, UBQ, Tubulin, RPL32, GAPDH, EF1A*	Developmental stage, tissue	*geNorm, Normfinder, BestKeeper, ΔC_*t*_ method, RefFinder*	Rodrigues et al., 2017
**LEPIDOPTERA**				
*Danaus plexippus*	*28S, 18S, EF1A, GAPDH, NADH, CypA, V-ATPase A, RPS5, RPL32*	Developmental stage, tissue, sex, temperature, photoperiod, dsRNA exposure	*geNorm, Normfinder, BestKeeper, ΔC_*t*_ method, RefFinder*	Pan et al., 2015a
*Chilo suppressalis*	*18S, Actin, α-tubulin, EF1A, Histone 3, RPS11, NADH, UBI, HSP60*	Tissue, organ, temperature	*geNorm, Normfinder, BestKeeper, ΔC_*t*_ method*	Xu et al., 2017
	*Actin A3, Actin A1, GAPDH, G3PDH, E2F, RPL32*	Developmental stage, tissue	*geNorm, NormFinder, stability index, ΔCt analysis*	Teng et al., 2012
*Spodoptera litura*	*EF1A, GAPDH, RPS3, RPL10, Actin, β-FTZ-F1, UCCR, ArgK*	Developmental stage, tissue, population, temperature, insecticide, diet, starvation	*geNorm, Normfinder, BestKeeper, ΔC_*t*_ method*	Lu et al., 2013
*Spodoptera exigua*	*Actin1, Actin2, EF1A, EF2, GAPDH, RPL10, RPL17, SOD, α-tubulin, 18S*	Developmental stage, tissue, sex	*geNorm, NormFinder, BestKeeper*	Zhu et al., 2014
	*Actin A3, Actin A1, GAPDH, G3PDH, E2F, RPL32*	Developmental stage, tissue	*geNorm, NormFinder, stability index, ΔCt analysis*	Teng et al., 2012
*Helicoverpa armigera*	*18S, 28S, Actin1, Actin2, α-tubulin, β-tubulin, GAPDH, EF1A, RPL13, RPS15, RPL27, RPL32*	Developmental stage, tissue, virus, insecticide, temperature	*geNorm, Normfinder, BestKeeper, ΔC_*t*_ method, RefFinder*	Zhang et al., 2015
	*β-tubulin, TATA, RPS15, HSP90, GAPDH, RPL28, ArgK, GST, Actin*	Developmental stage, mechanical injury, temperature, starvation, photoperiod	*geNorm, Normfinder, BestKeeper, ΔC_*t*_ method*	Shakeel et al., 2015
	*18S, β-tubulin, EF1A, GAPDH, Actin*	Developmental stage, dsRNA exposure	*geNorm, Normfinder, BestKeeper*	Chandra et al., 2014
*Sesamia inferens*	*18S, EF1A, GAPDH, RPS13, RPS20, tubulin, Actin*	Developmental stage, tissue, sex, temperature	*geNorm, Normfinder, BestKeeper, ΔC_*t*_ method, RefFinder*	Sun et al., 2015
*Plutella xylostella*	*18S, Actin, GAPDH, RPL32, RPS13, EF1A, RPS20, RPS23*	Development stage, tissue, population, temperature, photoperiod, insecticide, mechanical injury	*geNorm, Normfinder, BestKeeper, ΔC_*t*_ method*	Fu et al., 2013
	*Actin A3, Actin A1, GAPDH, G3PDH, E2F, RPL32*	Developmental stage, tissue	*geNorm, NormFinder, stability index, ΔCt analysis*	Teng et al., 2012
*Bombyx mori*	*Actin A3, Actin A1, GAPDH, G3PDH, E2F, RPL32*	Developmental stage, tissue	*geNorm, NormFinder, stability index, ΔCt analysis*	Teng et al., 2012
	*Actin1, Actin3, GAPDH, TIF-4A*	Virus, temperature	*ΔC_*t*_ method*	Guo et al., 2016
*Cryptophlebia peltastica*	*Actin, EF1A, α-tubulin, ArgK, CO1, Enolase*	Tissue	*geNorm, Normfinder, BestKeeper*	Ridgeway and Timm, 2015
*Cydia pomonella*	*Actin, EF1A, α-tubulin, ArgK, CO1, Enolase*	Tissue	*geNorm, Normfinder, BestKeeper*	Ridgeway and Timm, 2015
*Thaumatotibia leucotreta*	*Actin, EF1A, α-tubulin, ArgK, CO1, Enolase*	Tissue, temperature, virus	*geNorm, Normfinder, BestKeeper*	Ridgeway and Timm, 2015
*Gynaephora*	*18S, 28S, Actin1, Actin2, ArgK, Cyclin A, EF1A, GAPDH, RPL10, RPL27, RPL28, RPS15, RPS13, RPS2, Troponin C, β-tubulin, α-tubulin*	Population	*geNorm, Normfinder, BestKeeper, ΔC_*t*_ method, RefFinder*	Zhang et al., 2017
*Bicyclus anynana*	*Actin, EF1A, FK506, GAPDH, RPL40, V-ATPase H, RPS8, RPS18, HSP20, TATA, eIF2, G6PDH*	Developmental stage, tissue, sex, diet	*geNorm, Normfinder*	Arun et al., 2015
*Thitarodes armoricanus*	*18S, Actin, β-tubulin, GAPDH, G6PDH, EF2, EIF4A, RPL13*	Developmental stage, tissue, temperature, fungal infection, diet	*geNorm, Normfinder, BestKeeper*	Liu et al., 2016
*Heliconius numata*	*Actin, Annexin, EF1A, FK506BP, PolyABP, UBQ, RPL3, RPS3A, Tubulin*	Developmental stage	*geNorm, Normfinder, BestKeeper*	Piron Prunier et al., 2016
*Musca domestica*	*18S, Actin, EF1A, RPS18, GAPDH*	Developmental stage, mechanical injury, bacterial challenge	*geNorm, Normfinder, BestKeeper*	Zhong et al., 2013
**HEMIPTERA**				
*Bemisia tabaci*	*HSP40, HSP20, HSP70, HSP90, V-ATPase A, RPL29, EF1A, SDHA, Actin, PPIA, GAPDH, Myosin L, NADH, γ-tubulin*	Biotype, virus	*geNorm, Normfinder, BestKeeper, ΔC_*t*_ method, RefFinder*	Lü et al., 2017
	*18S, Actin, HSP20, HSP40, HSP70, HSP90, γ-tubulin, RPL29, SDHA, Flavoprotein, GAPDH, EF1A, PPIA, NADH, Myosin L, V-ATPase A*	Developmental stage, tissue, virus, biotype, photoperiod, temperature, insecticide	*geNorm, NormFinder*	Li et al., 2013
	*18S, Actin, a-tubulin, EF1A, GAPDH, RPL13, Cyclophilin1, TATA*	Insecticide	*geNorm, NormFinder, RefFinder*	Liang et al., 2014
	*Actin, GAPDH, GST, RPL32, SDHA, TATA, UBQ, a-tubulin*	Developmental stage, organ, insecticide, bacterial challenge	*geNorm, NormFinder*	Su et al., 2013
	*18S, GST, Actin, GAPDH, β-tubulin, a-tubulin, RPL13, EF1A*	Temperature	*geNorm, Normfinder, BestKeeper*	Dai et al., 2017
	*Actin, EF1A, GAPDH, RPL13, a-tubulin, Cyclophilin1*	Developmental stage, tissue, temperature	*geNorm, Normfinder, BestKeeper*	Collins et al., 2014
*Acyrthosiphon pisum*	*18S, 28S, 16S, Actin, EF1A, TATA, RPL12, β-tubulin, NADH, v-ATPase A, SDHB*	Developmental stage, temperature	*geNorm, Normfinder, BestKeeper, ΔC_*t*_ method, RefFinder*	Yang C. et al., 2014
*Lipaphis erysimi*	*16S, SDHB, Actin, EF1A, RPL13, RPS18, RPL27, RPL29, β-tubulin, GAPDH, ArgK*	Developmental stage, temperature, starvation, diet, glucosinolate	*geNorm, Normfinder, BestKeeper, ΔC_*t*_ method*	Koramutla et al., 2016
*Aphis glycines*	*SDFS, EF1A, Helicase, GAPDH, RPS9, TATA, UBQ*	Developmental stage, tissue, host plant	*geNorm, NormFinder*	Bansal et al., 2012
*Aphis craccivora*	*18S, 12S, EF1A, RPL11, V-ATPase D, RPL14, RPS8, RPS23, NADH, HSP70*	Developmental stage, temperature	*geNorm, Normfinder, BestKeeper, ΔC_*t*_ method, RefFinder*	Yang et al., 2015b
*Aphis gossypii*	*18S, 28S, Actin, GAPDH, EF1A, RPL7, α-tubulin, TATA*	Developmental stage, population, temperature, diet	*geNorm, Normfinder, BestKeeper, ΔC_*t*_ method*	Ma et al., 2016
*Myzus persicae*	*18S, Actin, RPL27, RPL7, β-tubulin, GAPDH, Acetylcholinesterase, EF1A, RPL32*	Development stage, tissue, host plant, wing dimorphism, photoperiod, temperature, insecticide	*geNorm, Normfinder, BestKeeper, ΔC_*t*_ method, RefFinder*	Kang et al., 2017
*Rhopalosiphum padi*	*18S, EF1A, Actin, GAPDH*	Wing dimorphism, virus	*geNorm, Normfinder, BestKeeper*	Wu et al., 2014
*Megoura viciae*	*RPL3, NADH, SDHA, RPS9, TATA, Actin, β-tubulin, UBQ*	Developmental stage	*geNorm, Normfinder, BestKeeper, ΔC_*t*_ method, RefFinder*	Cristiano et al., 2016
*Toxoptera citricida*	*18S, Actin, EF1A, GAPDH, α-tubulin, β-tubulin, RNAP II*	Developmental stage, wing dimorphism, temperature, starvation, UV irradiation	*geNorm, Normfinder, BestKeeper, ΔC_*t*_ method, RefFinder*	Shang et al., 2015
*Diuraphis noxia*	*Actin, RPL27, RPL9, RPL5, EF1A*	Host plant	*geNorm, Normfinder, BestKeeper*	Sinha and Smith, 2014
*Diaphorina citri*	*EF1A, Actin, α-tubulin, GAPDH, RPL7, RPL17*	Developmental stage, host plant	*geNorm, Normfinder, BestKeeper, ΔC_*t*_ method, RefFinder*	Bassan et al., 2017
*Toxoptera citricida*	*18S, EF1A, α-tubulin, β-tubulin, Actin, GAPDH, RNAP II*	Developmental stage, wing dimorphism, temperature, starvation, UV irradiation	*geNorm, Normfinder, BestKeeper, ΔC_*t*_ method, RefFinder*	Shang et al., 2015
*Rhodnius prolixus*	*Actin, α-tubulin, GAPDH, GST, G6PDH, SDHA, SP, EIF1A*	Developmental stage, aging, nutrition	*geNorm, Normfinder*	Omondi et al., 2015
*Rhodnius prolixus*	*18S, GAPDH, Actin, α-tubulin, RPL26*	Tissue, diet, virus	*geNorm, Normfinder, BestKeeper*	Paim et al., 2012
	*18S, EF1A, GAPDH, HSP70, Actin, Elav, MIP*	Organ, *Trypanosoma cruzi* infection	*geNorm, Normfinder*	Majerowicz et al., 2011
*Nilaparvata lugens*	*18S, Actin 1, Muscle actin, RPS11, RPS15, α-tubulin, EF1*Δ*, ArgK*	Developmental stage, tissue, population, temperature, insecticide, diet, starvation	*geNorm, Normfinder, BestKeeper, ΔC_*t*_ method*	Yuan et al., 2014
	*18S, Actin, α-tubulin, β-tubulin, EF1A, ETIF1*	Host plant, population	*geNorm, Normfinder, BestKeeper*	Wang W. X. et al., 2014
*Sogatella furcifera*	*18S, Actin, EF1A, α-tubulin, GAPDH, UBQ, RPS18, RPL9, RPL10*	Developmental stage, virus, tissue, temperature	*geNorm, Normfinder, BestKeeper, ΔC_*t*_ method, RefFinder*	An et al., 2016
*Euscelidius variegatus*	*18S, Actin, ATP synthase β, GAPDH, Tropomyosin*	Phytoplasma infection	*geNorm, Normfinder, BestKeeper*	Galetto et al., 2013
*Macrosteles quadripunctulatus*	*18S, Actin, ATP synthase β, GAPDH, Tropomyosin*	Phytoplasma infection	*geNorm, Normfinder, BestKeeper*	Galetto et al., 2013
*Ericerus pela*	*Actin1, Actin2, α-tubulin, β-tubulin1, β-tubulin2, SDHA1, SDHA2, SDHA3, RNAP II, RPL50-1, RPL50-2, RPL15, UBQ1, UBQ2, Myosin*	Developmental stage, tissue, temperature	*geNorm, Normfinder, RefFinder*	Yu et al., 2016
*Bactericera cockerelli*	*Actin, EF1A, Ferritin, GAPDH, RPL5, RPS18*	Developmental stage, tissue, Lso haplotype B infection	*geNorm, Normfinder, BestKeeper*	Ibanez and Tamborindeguy, 2016
*Cimex lectularius*	*α-tubulin, β-tubulin, RPL18, Actin, EF1A, GAPDH, SYN, UBQ*	Developmental stage, tissue, insecticide	*geNorm, Normfinder, BestKeeper*	Mamidala et al., 2011
*Delphacodes kuscheli*	*Actin, α-tubulin, GAPDH, EF1A, RPS18, UBQ*	Virus	*geNorm, Normfinder, BestKeeper*	Maroniche et al., 2011
*Phenacoccus solenopsis*	*Actin, RPL32, β-tubulin, α-tubulin, GAPDH, SDHA*	Developmental stage, host plant, temperature, population	*geNorm, Normfinder, RefFinder*	Arya et al., 2017
*Halyomorpha halys*	*RPS26, EF1A, UBQ, FAU, ARF, Actin, GUS, TATA, TIF6, RPL9*	Developmental stage, tissue, dsRNA exposure, starvation	*geNorm, Normfinder, BestKeeper, RefFinder*	Bansal et al., 2016
**DIPTERA**				
*Lucilia cuprina*	*18S, 28S, Actin, GST1, AChl, Per55, aE7, PKA, β-tubulin, GAPDH, RPLPO*	Developmental stage	*geNorm, Normfinder*	Bagnall and Kotze, 2010
*Lucilia sericata*	*18S, 28S, Actin, β-tubulin, RPS3, RPLP0, EF1A, PKA, GAPDH, GST1*	Naïve and immune-challenged larvae, tissue	*geNorm, Normfinder*	Baumann et al., 2015
*Liriomyza trifolii*	*18S, Actin, ArgK, EF1A, GAPDH, Histone 3, RPL32, α-tubulin, CAD*	Developmental stage, temperature, sex	*geNorm, Normfinder, BestKeeper, ΔC_*t*_ method, RefFinder*	Chang et al., 2017
*Drosophila melanogaster*	*18S, Actin, EF1A, Mnf, RPS20, RPL32, α-tubulin*	Mechanical injury, temperature, diet	*geNorm, Normfinder, BestKeeper*	Ponton et al., 2011
	*GAPDH, α-tubulin, RPL32, RPL13, EF1A, SDHA, GST1, Cyp1, Tyrosine-3-monooxygenase, exba, Actin, Su (Tpl), Faf, CG13220, Robl, Rap2l, HMBS, RNAP II, Nrv2, Elav, Appl*	Aging- or neurodegeneration-related sample	*SAS*	Ling and Salvaterra, 2011
	*Actin, β-tubulin, GAPDH, RPL32,TATA, eIF2*	Imaginal disk	*geNorm, Normfinder*	Matta et al., 2011
*Drosophila suzukii*	*Actin, GAPDH, RPL18, RPS3, ArgK, EF1β, NADH, HSP22, α-tubulin, TATA*	Developmental stage, tissue, population, photoperiod, temperature	*geNorm, Normfinder, BestKeeper, RefFinder*	Zhai et al., 2014
*Bactrocera dorsalis*	*18S, Actin1, Actin2, Actin3, Actin5, GAPDH, G6PDH, α-tubulin, β-tubulin, EF1A*	Tissue	*geNorm, Normfinder*	Shen et al., 2010
	*18S, β-tubulin, RPL13, GAPDH, EF1A, SDHA, α-tubulin, Actin, RNAP II*	β-Cypermethrin, tissue	*geNorm, Normfinder*	Shen et al., 2013
*Anastrepha obliqua*	*Actin, β-tubulin, GAPDH, RPL18, RPS17, Syntaxin, Troponin C*	Developmental stage	*Normfinder, BestKeeper, RefFinder*	Nakamura et al., 2016
*Bactrocera (Tetradacus) Minax*	*18S, 28S, GAPDH, α-tubulin, β-tubulin, Actin, G6PDH, RPL32, EF1A, EF1β*	Developmental stage, temperature, *γ-*irradiation	*geNorm, Normfinder, RefFinder*	Lü et al., 2014
*Bradysia odoriphaga*	*Actin, EF1A, UBQ, RSP5, α-tubulin, GAPDH, RPS18, RPL18, SDHA, RPL28, RPS13, RPS15*	Developmental stage, temperature, insecticide, photoperiod, diet, population	*geNorm, RefFinder*	Shi et al., 2016
*Aedes aegypti*	*Actin, EF1A, α-tubulin, RPL8, RPL32, RPS17, GAPDH*	Developmental stage	*geNorm, BestKeeper, NormFinder*	Dzaki et al., 2017
*Chrysomya megacephala*	*Actin, RPL8, GAPDH, EF1A, α-tubulin, β-tubulin, TATA, 18S, RPS7*	Developmental stage, tissue, drug, heavy metal, diet	*RefFinder*	Wang et al., 2015
*Ceratitis capitata*	*RPL19, TATA, Ultrabithorax, GAPDH, α-tubulin, β-tubulin, 14-3-3zeta, RNA polymerase II, Actin3*	Developmental stage, tissue, body part	*geNorm, Normfinder, BestKeeper, RefFinder*	Sagri et al., 2017
*Bactrocera oleae*	*RPL19, TATA, Ultrabithorax, GAPDH, α-tubulin, β-tubulin, 14-3-3zeta, RNAP II, Actin3*	Developmental stage, tissue, body part	*geNorm, Normfinder, BestKeeper, RefFinder*	Sagri et al., 2017
**HYMENOPTERA**				
*Solenopsis invicta*	*RPL18, EF1β, Actin, GAPDH, TATA*	Developmental stage, tissue, caste	*geNorm, Normfinder, BestKeeper, RefFinder*	Cheng et al., 2013
*Apis mellifera*	*Actin, GAPDH, α-tubulin, RPS18, GST1, RPL32, UBQ, RPL13, HMBS, SDHA, TATA*	Bacterial challenge	*geNorm, Normfinder, BestKeeper*	Scharlaken et al., 2008
	*GAPDH, RPL32, EF1A*	Aging	*geNorm, Normfinder, BestKeeper*	Reim et al., 2013
	*RPL19, RPL27, RPL10, RPL12, RPS18, GAPDH, EIF5A, Pontin, Proteasome, NAPK, U2af38, Pros54, DCAF13, ROSM1, NADH*	Development time	*geNorm, Normfinder, BestKeeper*	Cameron et al., 2013
*Bombus terrestris*	*ELF1A, PPIA, RPL23, TATA, polyubiquitin*	Virus	*geNorm, Normfinder*	Niu et al., 2014
	*ArgK, EF1A, PLA2, α-tubulin, GAPDH, Actin, RPL13*,	Tissue	*geNorm, Normfinder*	Hornáková et al., 2010
*Bombus lucorum*	*ArgK, EF1A, PLA2, α-tubulin, GAPDH, Actin, RPP2*	Tissue	*geNorm, Normfinder*	Hornáková et al., 2010
*Lysiphlebia japonica*	*18S, Actin, β-tubulin, RPL18, ArgK, EF1A, TATA, PRII, RPL27, RPS18, DIMT, PPI*	Developmental stage, tissue, sex, diet	*geNorm, Normfinder, BestKeeper*	Gao et al., 2017
**THYSANOPTERA**				
*Frankliniella occidentalis*	*28S, 18S, Actin, α-tubulin, EF1A, V-ATPase A, NADH, HSP60, HSP70, HSP90, RPL32*	Virus	*geNorm, Normfinder, BestKeeper, ΔC_*t*_ method, RefFinder*	Yang et al., 2015a
	*18S, Actin, α-tubulin, EF1A, GAPDH, Histone 3, RPL32*	Developmental stage, temperature	*geNorm, Normfinder, BestKeeper, RefFinder*	Zheng et al., 2014
**BLATTODEA**				
*Diploptera punctata*	*Actin, α-tubulin, GAPDH, Armadillo, RPL32, SDHA, EF1A, Annexin IX*	Tissue	*geNorm, Normfinder*	Marchal et al., 2013
**ORTHOPTERA**				
*Chortoicetes terminifera*	*18S, GAPDH, Actin, α-tubulin, RPL32, EF1A, Annexin IX, SDHA*	Solitarious and gregarious phase, isolated or crowded condition, short-term crowding	*geNorm, Normfinder*	Chapuis et al., 2011
*Schistocerca gregaria*	*GAPDH, Actin, α-tubulin, UBI, EF1A, RPL32, CGI3220*	Developmental stage	*geNorm, Normfinder*	Van Hiel et al., 2009
*Locusta migratoria*	*18S, Ach, Actin, Chtinase2, EF1A, RPL32, HSP70, α-tubulin, RPL32, SDHA, GAPDH, Histone*	Developmental stage, tissue, insecticide, temperature, starvation	*geNorm, Normfinder, BestKeeper, ΔC_*t*_ method*	Yang Q. et al., 2014
**SIPHONAPTERA**				
*Ctenocephalides felis*	*18S, 28S, Actin, Muscle actin, EF1A, GAPDH, HSP22, NADH, RPL19, α-tubulin*	Developmental stage, sex, diet, insecticide	*geNorm, Normfinder, BestKeeper*	Mcintosh et al., 2016
**PSOCOPTERA**				
*Liposcelis bostsrychophila*	*18S, Actin1, Actin2, α-tubulin, GAPDH*	Developmental stage, insecticide	*geNorm*	Jiang et al., 2010

* *ADP-ribosylation factor (ARF), β-actin (Actin), elongation factor 1 α (EF1A), glyceralde hyde-3-phosphate dehydrogenase (GAPDH), glucose-6-phosphate dehydrogenase (G6PDH), arginine kinase (ArgK), cyclophilins A (CypA), vacuolar-type H^+^-ATPase subunit A (V-ATPase A), 16S ribosomal RNA (16S), 12S ribosomal RNA (12S), 28S ribosomal RNA (28S), 18S ribosomal RNA (18S), ribosomal protein S (RPS), ribosomal protein L (RPL), ribosomal protein P2 (RPP2), heat shock protein (HSP), NADH dehydrogenase subunit 2 (NADH), succinate dehydrogenase complex subunit A (SDHA), peptidylprolyl isomerase A (PPIA), myosin light chain (Myosin L), glutathione S-transferase (GST), succinate dehydrogenase flavoprotein subunit (SDFS), ubiquitin-conjugating protein (UBQ), RNA polymerase II large subunit (RNAP II), superoxide dismutase (SOD), cAMP-dependent protein kinase A (PKA), acidic ribosomal phosphoprotein PO (RPLPO), acetylcholinesterase (AChl), peritrophin-55 (Per55), alpha esterase 7 (aE7), ADP-ribosylation factor (ARF), porphobilinogen deaminase (HMBS), cytochrome oxidase subunit 1 (CO1), cytochrome P450 CYP6 (CYP6), embrionic lethal abnormal vision (Elav), major intrinsic protein (MIP), ubiquinol-cytochrome c reductase (UCCR), dimethyladenosine transferase (DIMT), peptidylprolyl isomerase (PPI), FK 506 binding protein (FK506), translation initiation factor eIF2 alpha (eIF2), acetyl-CoA hydrolase (Ach), translation elongation factor 2 (EF2), translation initiation factor 4A transporter-like (EIF4A), carbamoyl phosphate synthase (CAD), E2F transcription factor 4-like protein (E2F), FK506 binding protein (FK506BP), polyA binding protein (polyABP), forkhead domain 68A (Mnf), beta amyloid protein precursor-like (Appl), embryonic lethal abnormal vision (Elav), Na+/K+ ATPase (Nrv2), hydroxymethylbilane synthase (HMBS), ras-associated protein 2-like (Rap2l), roadblock-type 2 (Robl), acidic phospholipase A2 (PLA2), ubiquitin-like protein FUBI (FAU), β-glucuronidase (GUS), serine protease (SP), proteasome subunit beta type-7-like (Proteasome), nucleoside diphosphate kinas (NDPK), U2 small nuclear riboprotein auxiliary factor 38 (U2af38), proteasome 54kD subunit (Pros54), DDB1- and CUL4-associated factor 13-like (DCAF13), reactive oxygen species modulator 1-like (ROSM1)*.

In order to fill this gap and provide molecular biologists with informative guidance on selecting the reference genes to customize their RT-qPCR experiments, this present review summarizes the current trends in reference gene selection for RT-qPCR normalization in gene expression studies performed on insects between 2008 and 2017 (Table 1). Specifically, the insect species, reference genes, experimental conditions, analysis tools, and publication year have been summarized. Furthermore, the relationships between the numbers of the reference genes, experimental factors, analysis tools, and publication date (year) were investigated by linear regression. We hoped that our meta-analysis would be of great help for researchers that plan gene expression studies in insects, especially the non-model ones, as it provides a summary of appropriate reference genes for expression studies, considers the optimal number of reference genes, and reviews average numbers of experimental factors and analysis tools per study.

## Number of relevant studies in insects that utilized expression levels of reference genes for normalization of RT-qPCR data

**Figure 1:**
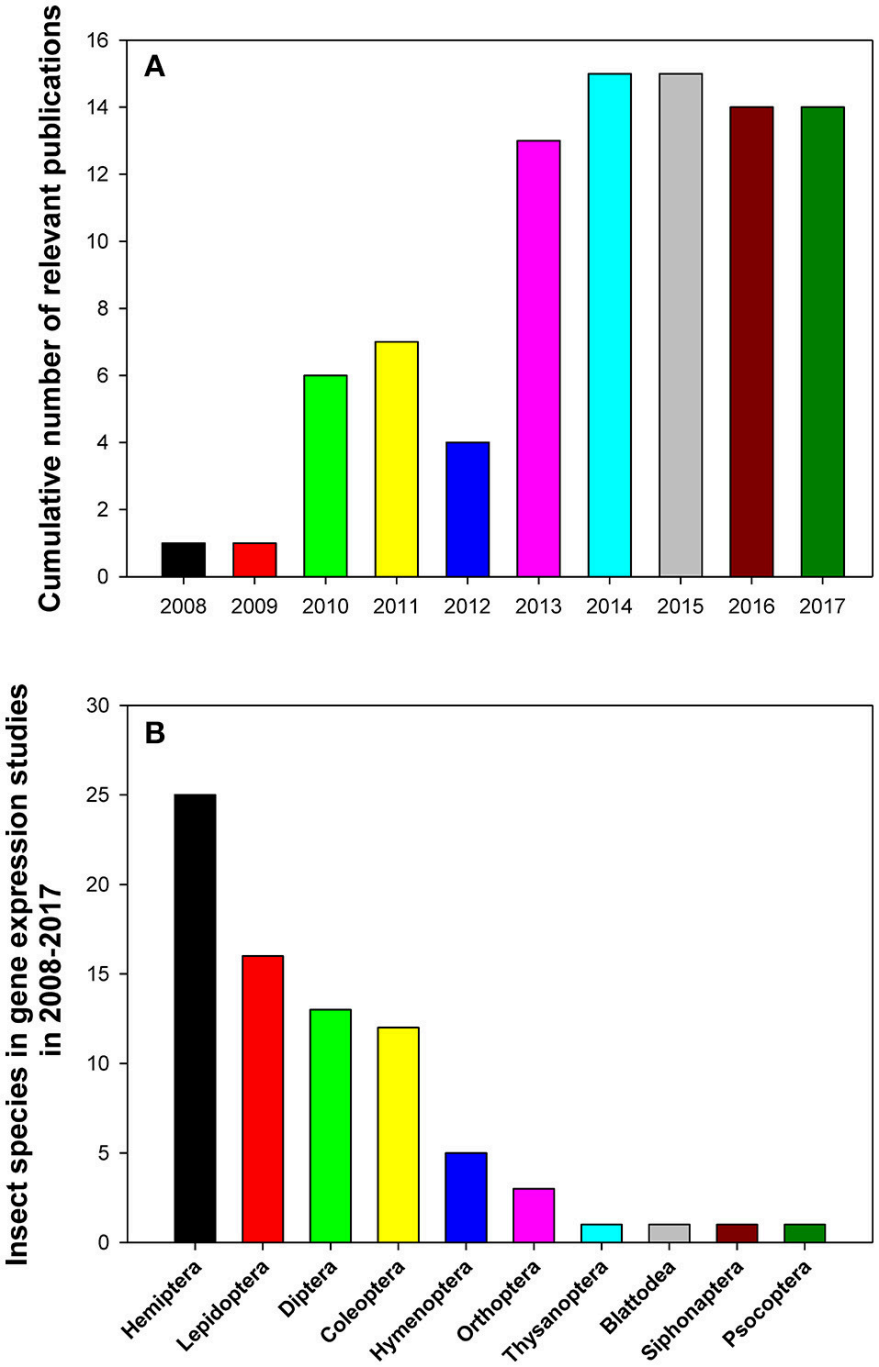
Cumulative numbers of relevant publications **(A)** and distribution of insect species belonging to different taxonomic orders **(B)** in relevant gene expression studies performed in 2008–2017 that utilized expression levels of reference genes to normalize RT-qPCR data.

The relevant publications that analyzed reference gene expression in insects in 2008–2017 are summarized in Table 1. All data were extracted from databases such as https://www.ncbi.nlm.nih.gov/pubmed, https://scholar.google.com/, https://link.springer.com/, http://onlinelibrary.wiley.com/, and https://www.sciencedirect.com/ using the following search terms: (“internal control genes” OR “reference genes” OR “housekeeping genes”) AND (“qPCR” OR “quantitative PCR” OR “qRT-PCR” OR “RT-qPCR”) occurring in the Title*/*Abstract. Additionally, we also curated relevant papers that came to our attention independently but were not uncovered by the above search algorithm. We found and curated 90 representative papers published in 36 journals. The top five journals by the number of published studies on gene expression in insects were PLoS One (26/90), Scientific Reports (9/90), Journal of Economic Entomology (6/90), Journal of Insect Science (5/90), and BMC Research Notes (4/90; Table 1). These papers were mainly published between 2013 and 2017 with an average of 14 papers published over the last 5 years (Figure 1A). We can clearly see that open access journals provide the main platform for publications on this topic.

## Number of insect species that were analyzed for expression of reference genes

The 90 reviewed papers reported results of gene expression studies in 78 insect species in 100 separate experiments (Table 1). These insects were from 10 insect orders (Figure 1B). They predominantly belonged to the following four insect orders: Hemiptera (25 insect species), Lepidoptera (16 insect species), Coleoptera (12 insect species), and Diptera (13 insect species; Figure 1B). Some insects, such as *Bemisia tabaci* (Li et al., 2013; Su et al., 2013; Collins et al., 2014; Liang et al., 2014; Dai et al., 2017; Lü et al., 2017) and *Helicoverpa armigera* (Chandra et al., 2014; Shakeel et al., 2015; Zhang et al., 2015), which cause serious damage to crops, were investigated extensively and frequently. There were six and three papers, respectively, for the above-mentioned species that analyzed expression levels of reference genes and were published during the last 5 years.

## Distribution of the number of reference genes per study

**Figure 2:**
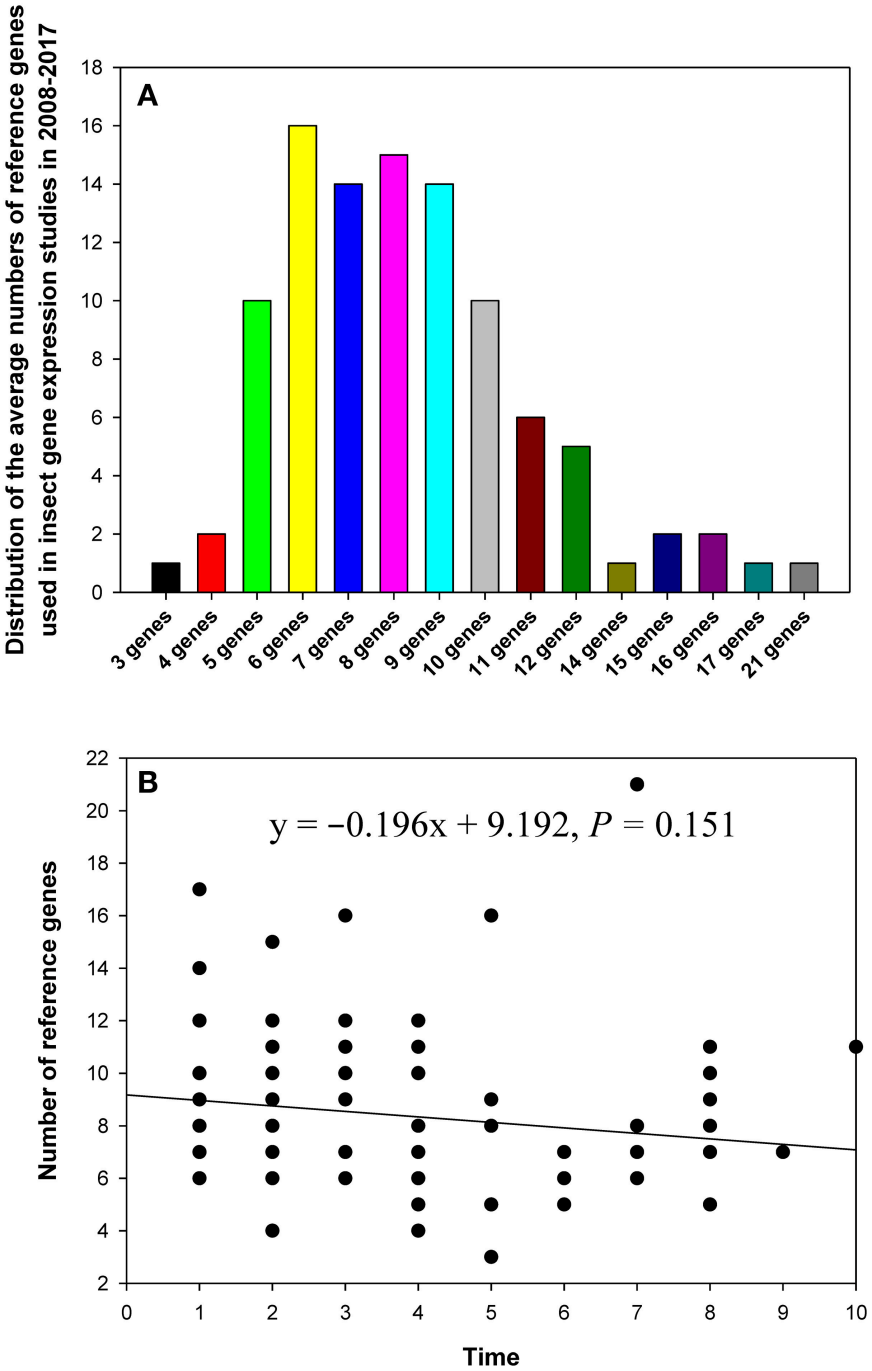
The distribution of the numbers of reference genes per study in relevant publications about gene expression in insects in 2008–2017 **(A)**, and the relationship between the number of reference genes and study publication date (year) fitted by linear regression **(B)**. The numbers 1–10 on the X-axis represent years from 2017 to 2008, respectively.

In the 90 papers, 3–21 reference genes were investigated per single study (Figure 2). In the majority of studies, the expression level of 5–10 reference genes was determined (Figure 2A). The breakdown of the papers that analyzed expression of multiple reference genes was as follows: five genes (10%), six genes (16%), seven genes (14%), eight genes (15%), nine genes (14%), and ten genes (10%). Recently, in some studies, more than 10 candidate reference genes were analyzed to provide more choices for expression level comparisons and normalization (Table 1). However, linear regression analysis did not reveal a significant correlation between the number of reference genes used in the study and its publication date (year; Figure 2B).

## Top 10 reference genes

**Figure 3:**
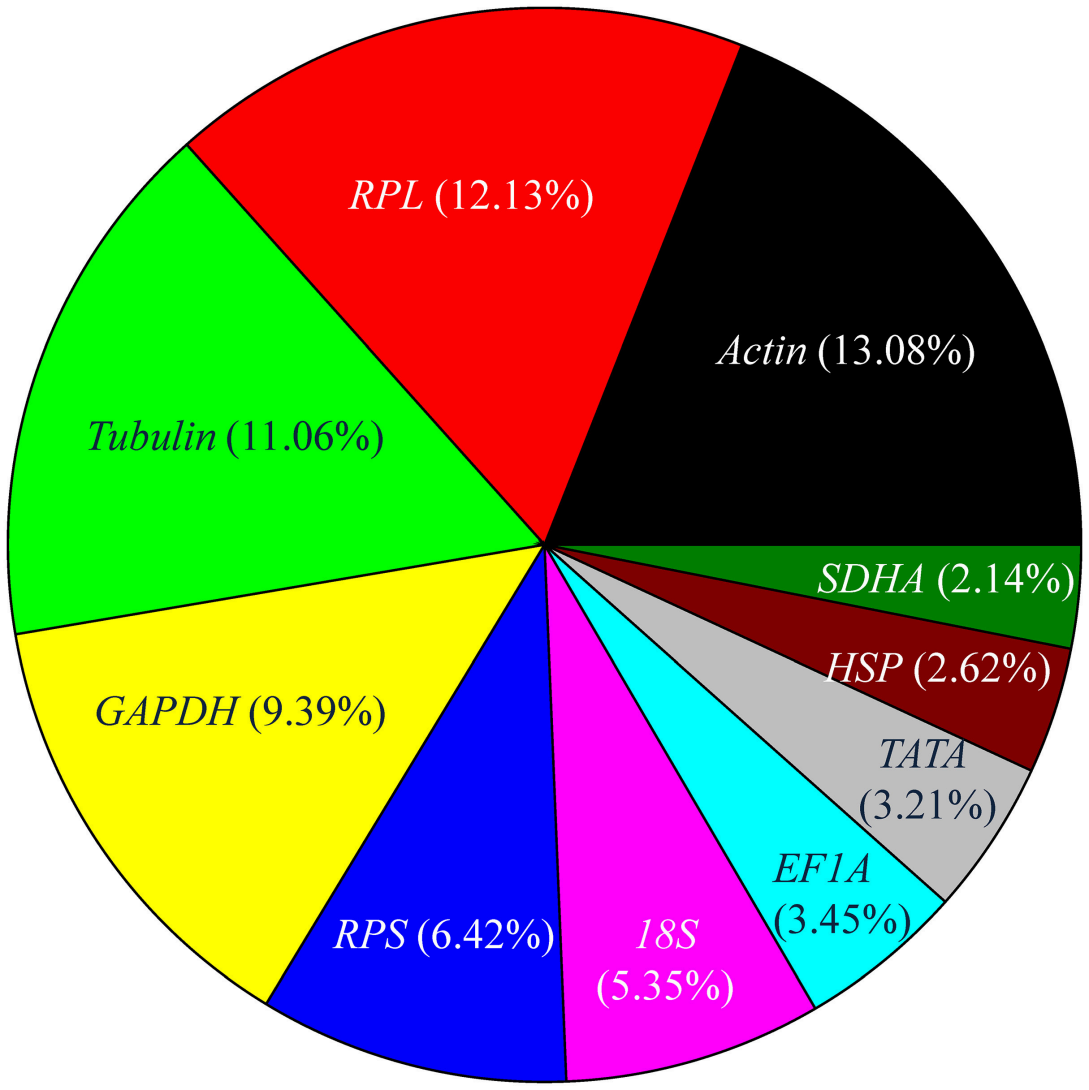
Frequency of the top 10 most popular reference genes in relevant insect gene expression studies performed during 2008–2017. *RPL* includes *RPL3, RPL4, RPL5, RPL7, RPL8, RPL9, RPL10, RPL11, RPL12, RPL13, RPL14, RPL15, RPL17, RPL18, RPL19, RPL22, RPL23, RPL26, RPL27, RPL28, RPL29, RPL32, RPL40*, and *RPL50*; *RPS* includes *RPS2, RPS3, RPS5, RPS6, RPS7, RPS8, RPS9, RPS11, RPS13, RPS15, RPS17, RPS18, RPS20, RPS23, RPS24, RPS26*, and *RPS27, Tubulin* includes α*-tubulin*, β*-tubulin*, and γ*-tubulin*; *HSP* includes *HSP20, HSP22, HSP40, HSP60, HSP70*, and *HSP90*.

In the set of curated 90 papers, the expression level of reference genes was determined for 841 times. The number of experiments that utilized top 10 most frequently used reference genes, including *Actin, RPL, Tubulin, GAPDH, RPS, 18S, EF1A, TATA, HSP*, and *SDHA*, are shown in Figure 3. *Actin*, which encodes a major structural protein, is expressed at various levels in many cell types. It is considered the ideal reference gene for RT-qPCR analysis and has been investigated most frequently (Figure 3). For example, previous studies have shown that the expression of *Actin* was the most stable among other reference genes across different developmental stages of many insects, including *Apis mellifera, Schistocerca gregaria, Drosophila melanogaster, Plutella xylostella, Chilo suppressalis, Chortoicetes terminifera, Liriomyza trifolii*, and *Diuraphis noxia* (Scharlaken et al., 2008; Van Hiel et al., 2009; Chapuis et al., 2011; Ponton et al., 2011; Teng et al., 2012; Sinha and Smith, 2014; Chang et al., 2017). Nonetheless, the expression of *Actin* was less stable in several insects, including those of the species, *Coleomegilla maculata, Coccinella septempunctata*, and *Hippodamia convergens* of the family Coccinellidae (Pan et al., 2015b; Yang et al., 2015c, 2016).

Ribosomal protein (RP), a principal component of ribosomes, is among the most highly conserved proteins across all life forms. The fraction of studies in which the expression level of *RPL* and *RPS* family genes was used as reference was 18.55%. Together, these genes were the most widely selected reference genes for expression studies in insects during the past 10 years. In most of these studies, RP-encoding genes were stable reference genes. For example, *RPS24* and *RPS18* were stable reference genes across different developmental stages and sex treatments of *C. maculata* (Yang et al., 2016); *RPS13* and *RPS23* were stable reference genes across different developmental stages of *P. xylostella* (Fu et al., 2013); whereas *RPL11, RPS8*, and *RPL14* were the three most stable reference genes across different developmental stages and under different temperature conditions of *Aphis craccivora* (Yang et al., 2015b). However, under some conditions, expression levels of RP-encoding genes may be unstable. For example, *RPS20* was the least stable gene in *P. xylostella* strains that were collected in different fields, grown under different temperatures, exposed to different photoperiods, or presented different insecticide susceptibility (Fu et al., 2013).

*Tubulin* (α*-tubulin*, β*-tubulin*, and γ*-tubulin*), which encodes cytoskeletal structure proteins, was ranked as the third most widely investigated reference gene (Figure 3). In many studies, the stability of *Tubulin* was variable under different treatments for the same species. For example, *a-tubulin* exhibits a stable expression in different tissues and sexes of *C. maculata*, whereas its expression was unstable across different developmental stages and following dsRNA treatments (Yang et al., 2015c).

*GAPDH* is another commonly used reference gene, ranked as the fourth most widely utilized reference gene (Figure 3). Occasionally, the stability of *GAPDH* expression was variable under different treatments within the same species. For example, *GAPDH* expression was not affected by tissue type, sex, photoperiod, or dsRNA treatment in *H. convergens*, but it varied across different developmental stages and at different temperatures (Pan et al., 2015b). *GAPDH* was a stable reference gene whose expression was not appreciably altered under different temperatures or by mechanical injury in different strains of *P. xylostella*; however, its expression was unstable across different developmental stages and was affected by photoperiod (Fu et al., 2013).

*18S* ribosomal RNA, a part of the ribosomal RNA, was ranked as the sixth most widely investigated reference gene (Figure 3). It was stably expressed throughout the vast majority of biotic and abiotic conditions in most studies that employed its expression level as reference (Table 1). However, it is generally acknowledged that the use of rRNA for normalization of RT-qPCR signals is problematic as rRNA forms a significant proportion of the total RNA pool (>80%), whereas mRNA accounts for a mere 3–5%, so the subtle changes in target gene expression levels may be potentially masked. With this in mind, it is much better to use the mRNA species of the ribosomal machinery, such as *RPL* and *RPS* genes, instead of rRNA.

Altogether, the expression level of *EF1A, TATA, HSP*, and *SDHA* genes was used as a reference in 11.42% of the experiments. These four genes transiently exhibited variable expression under different treatments in different insect species. For example, *EF1A* was the least stable reference gene in *A. craccivora* across different developmental stages and at different temperatures (Yang et al., 2015b). In contrast, *EF1A* was one of the best reference genes in *H. convergens* with its expression level being unaffected by three biological factors (developmental stage, tissue type, and sex) and three abiotic conditions (temperature, photoperiod, and dietary RNAi; Pan et al., 2015b).

## Distribution of the numbers of experimental factors studied

**Figure 4:**
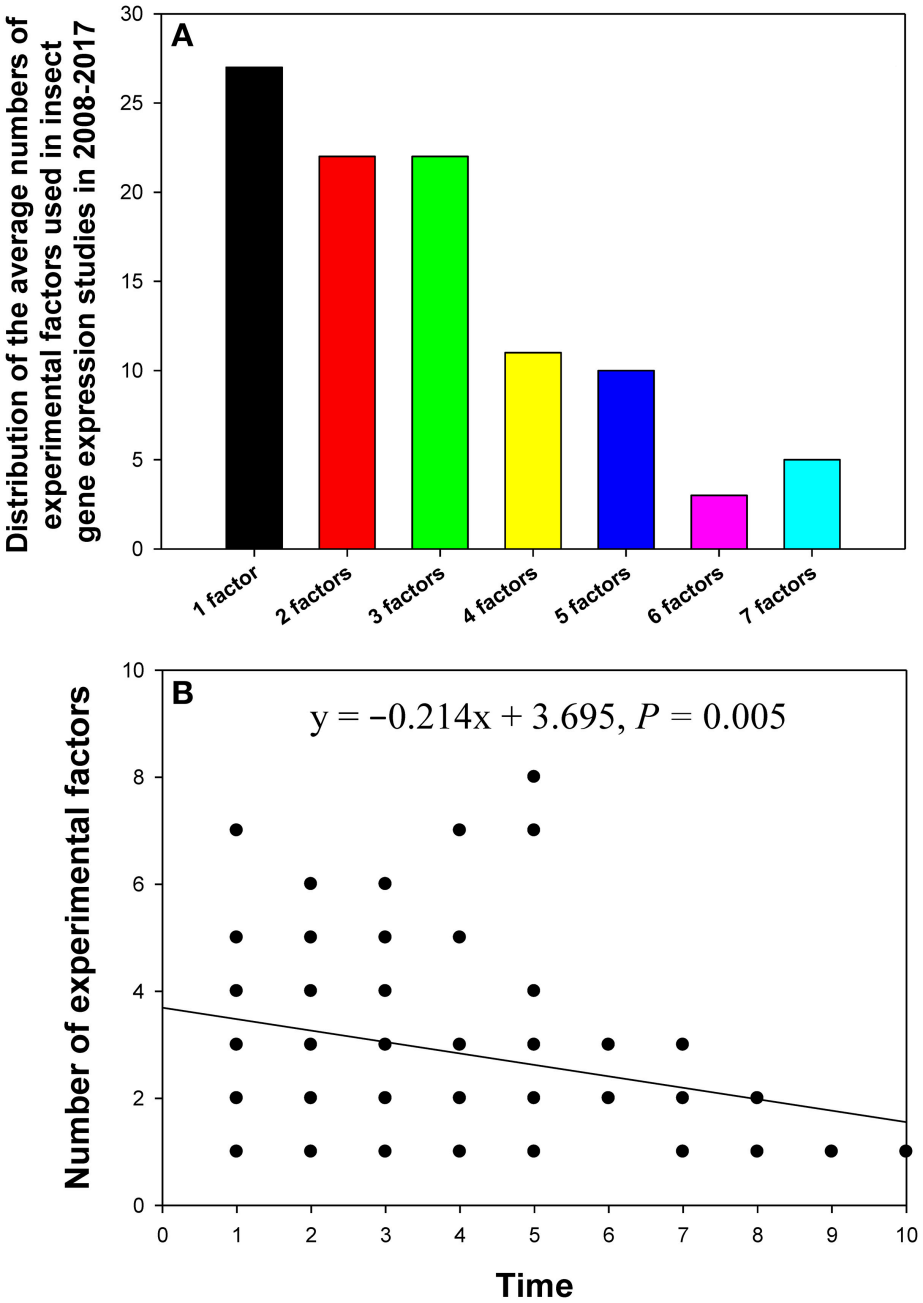
Distribution of the number of experimental factors in relevant insect gene expression studies performed during 2008–2017 **(A)**, and the relationship between the number of experimental factors per study and study publication date (year) investigated by linear regression **(B)**. The numbers 1–10 on the X-axis represent years from 2017 to 2008.

In the 90 papers, changes in the reference gene expression level were investigated under the influence of one to seven experimental factors. Most of these studies analyzed the influence of one (10%), two (16%), or three (14%) experimental factors (Figure 4A). The relationship between the number of experimental factors and study publication date (year) was investigated by linear regression. We found that the more recently the paper was published, the more experimental factors it tended to explore (Figure 4B).

## Top 10 experimental factors

**Figure 5:**
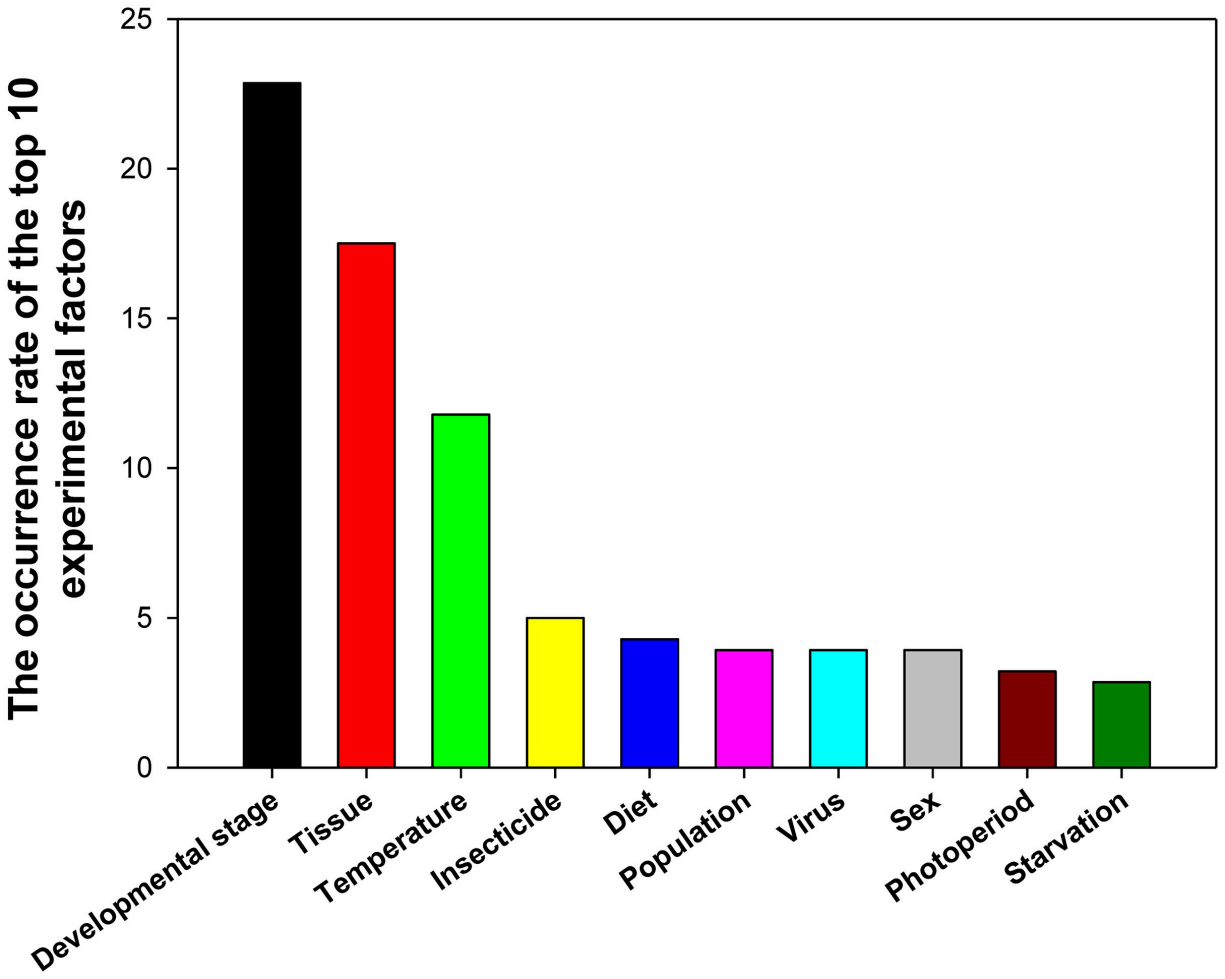
Frequency of top 10 experimental factors in relevant insect gene expression studies performed during 2008–2017.

A total of 39 experimental factors were investigated in these 90 papers, with the top 10 experimental factors (in the descending order) being developmental stage, tissue, temperature, insecticide, diet, population, virus, sex, photoperiod, and starvation (Figure 5).

RNA interference (RNAi) is a conserved mechanism whereby messenger RNA transcripts are targeted by small interfering RNAs in a sequence-specific manner, leading to downregulation of gene expression. During the past 20 years, RNAi has been widely used as a tool to investigate functions of insect genes (Zotti et al., 2018), whereas RT-qPCR is the method of choice to study gene expression in terms of its sensitivity and specificity. The genes that play important roles during insect metamorphosis and affect different tissues can serve as target genes for manipulations that kill the insect or retard its growth. This is why gene expression profiles are widely assessed at different developmental stages and in different tissues. The effect of these two factors on gene expression was investigated frequently with the use of reference gene expression levels in 22.86 and 17.50% of studies, respectively (Figure 5).

Insects are ectothermic organisms, and the body temperature of most insects is affected by changes in ambient temperature, ultimately influencing their growth, and development. Temperature was ranked as the third most widely investigated factor at 11.79% (Figure 5). We found that the numbers/kinds of reference genes under different temperatures varied in different insects. For instance, *GAPDH*, and *EF1A* were the best stable gene combinations in *Spodoptera litura* (Lu et al., 2013), while *RPS15*, β*-tubulin*, and *EF1A* were the most stable reference genes in *Nilaparvata lugens* (Yuan et al., 2014).

Many insects, including the 78 insect species summarized in this study have developed resistance to insecticides. Insecticide resistance presents as a major challenge for pest control. The molecular mechanisms underlying insecticide resistance are under intense scrutiny; RT-qPCR is an important technology for investigating the gene functions involved in insecticide resistance. Insecticides ranked as the fourth most widely investigated factor at 5.00% (Figure 5). We found that different reference genes were used in different insects to study the effect of various insecticide treatments. *RPS15* and *RPL32* were stably expressed reference genes in insecticide treatment experiments in *H. armigera* (Zhang et al., 2015); while *RPS11, EF1A*, and β*-tubulin* were the best choice in the insecticide-stressed *N. lugens* (Yuan et al., 2014). Different classes of insecticides have warranted different sets of reference genes to normalize target gene expression in *B. tabaci* (Liang et al., 2014).

Diet was ranked as the fifth most widely investigated factor at 4.29% (Figure 5). Different gene combinations were required for different diet conditions. For examples, *RPL10* and *GAPDH* were the most stable reference genes in *S. litura* that were reared on different diets (Lu et al., 2013); whereas, *Actin, RPS18*, and *RPS15* were the most stable reference genes among different diets in *Bradysia odoriphaga* (Shi et al., 2016), *Actin* and *18S* were the best reference gene combination for feeding assay experiments with *Aphis gossypii* (Ma et al., 2016).

Population, virus, and sex were all ranked as the sixth most widely investigated factor at 3.93%(Figure 5). Different reference gene combinations were suggested for the studies of each factor. For example, *RPL10* and *EF1A* were the most stable reference genes in *S. litura* collected from different locations (Lu et al., 2013), *EF1A, Actin*, and *GAPDH* were the more stable reference genes in *P. xylostella* (Fu et al., 2013). The combination of *Actin* and *EF1A* was very useful for experiments involving *A. gossypii* (Ma et al., 2016*)*. In addition, in viral infection experiments, different reference gene combinations were recommended for different insects. For example, *GAPDH, RPL27*, and β*-tubulin* was the best reference gene combination for nuclear polyhedrosis virus infection (Zhang et al., 2015), *HSP90* and *RPL29* were the most stable reference genes in *B. tabaci* when the whitefly carried the tomato yellow leaf curl virus and when it did not (Li et al., 2013). Moreover, in females and males, different reference gene combinations were recommended for different insects. For instance, *GAPDH* and *CypA* were most stable reference genes for *H. convergens* (Pan et al., 2015b), *HSP90* and *RP49* were the most stable ones for *Harmonia axyridis* (Yang et al., 2018), and *18S, EF1A*, and *GAPDH* were the best for gene expression normalization in *Sesamia inferens* (Sun et al., 2015).

Photoperiod and starvation ranked as the seventh and eighth most widely investigated factors at 3.21 and 2.86%, respectively (Figure 5). Different reference gene combinations were recommended for different insects for these two factors. For instance, under photoperiod stressed conditions, *GAPDH* and *CypA* were most stable reference genes in for *H. convergens* (Pan et al., 2015b), *EF1A* and *V-ATPase A* were the most stable ones for *Danaus plexippus* (Pan et al., 2015a), and *HSP90* and β*-tubulin* were the best reference genes for *H. armigera* (Shakeel et al., 2015). Under starvation conditions, *RPL28* and *RPS15* were the most stable reference genes for *H. armigera* (Shakeel et al., 2015), *RPS3* and *Actin* were the best reference genes for *S. litura* (Lu et al., 2013), and *RPS11, ArgK*, and *EF1A* were recommended for *N. lugens* (Yuan et al., 2014).

## Distribution of the number of analysis tools

**Figure 6:**
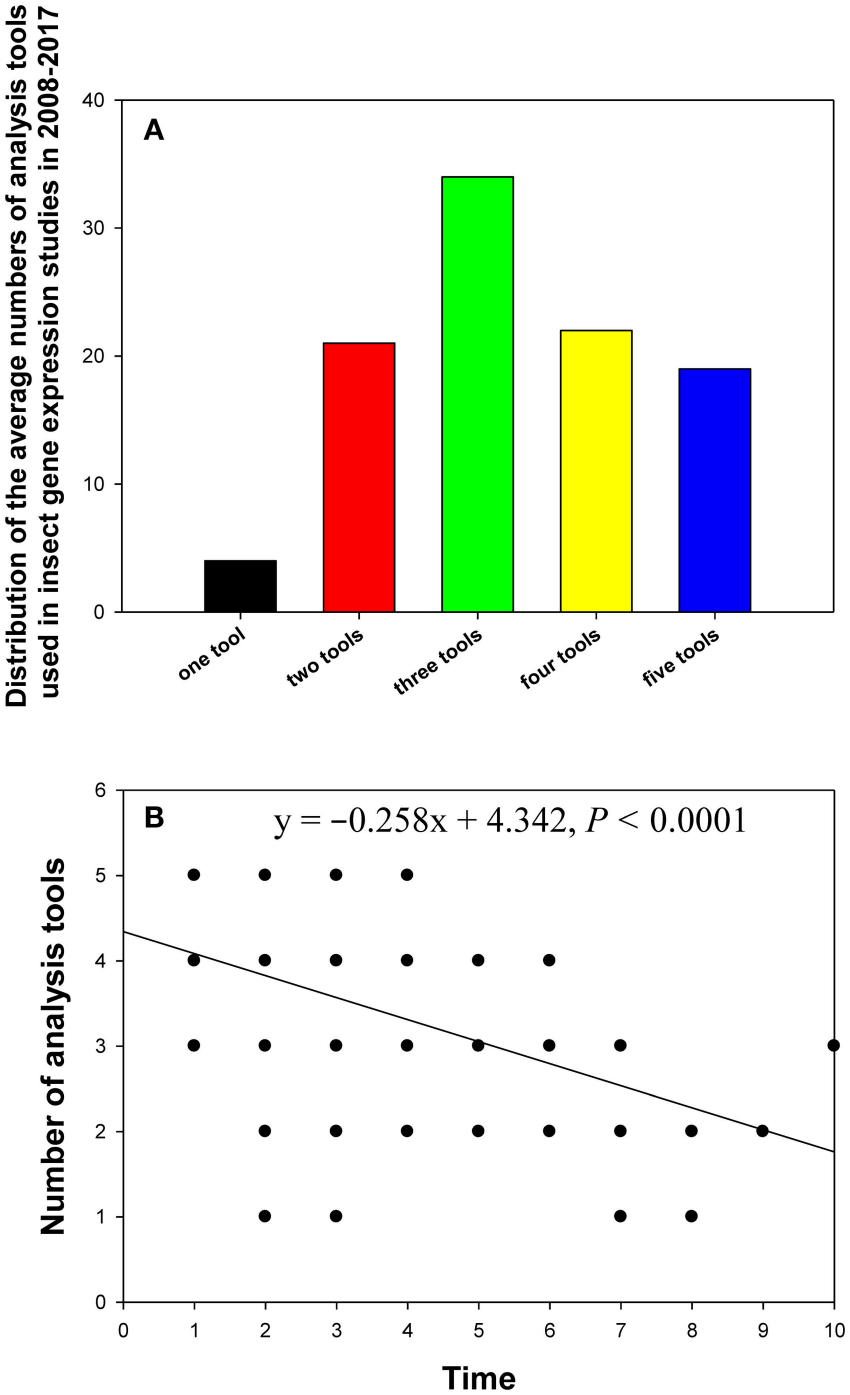
Distribution of the numbers of analysis tools in relevant insect gene expression studies performed during 2008–2017 **(A)**, and the relationship between the number of analysis tools per study and study publication date (year) investigated by linear regression **(B)**. The numbers 1–10 on the X-axis represent years from 2017 to 2008.

In the 90 papers, one to five analysis tools were used to evaluate gene expression stability, with one tool (4%) and three tools (34%) being the least and most frequently used variants in these studies, respectively (Figure 6A). Linear regression analysis showed that the more recently the paper was published, the more analysis tools it used (Figure 6B).

## Conclusions

Our review clearly suggests that no reference gene is universally stably expressed because variable expression levels even for the most popular reference genes have been observed under different circumstances in the same insect species or under the same experimental condition among different insects. In order to obtain reliable experimental data for the target gene, it is necessary to perform internal reference gene screening under specific experimental conditions. Given that the best internal reference genes in different species under different conditions often have large differences in expression, it may result in a multi-fold difference of target gene expression, or even false conclusion, if used improperly. For instance, the expression of *V-ATPase A* in the gut ranged from 7.7- to 22.4-fold higher than that in the carcass of *C. septempunctata* when normalized to the most- and least-stable sets of reference genes, respectively (Yang et al., 2016). Furthermore, the relative *hsp83* expression was noticeably variable when a less stable reference gene was used for RT-qPCR normalization in different tissues and developmental stages of *S. inferens*, whereas *hsp83* was uniformly expressed when stable reference genes were used for normalization (Sun et al., 2015). Therefore, better accuracy in gene expression analysis can promote the investigation of gene function. We strongly recommend that prior to each RT-qPCR experiment, the reference gene expression stability must be validated. Furthermore, multiple reference genes should be used to achieve the best results. This review should help researchers select the best reference genes and optimize their experiments to examine gene expression levels in insects, especially the non-model ones, in terms of the number of reference genes chosen, experimental factors manipulated, and the analysis tools used.

## Author contributions

HP and YZ conceived the topic of the review. HP, CY, and JL performed literature review analyzed the data. HP and CY wrote the manuscript.

### Conflict of interest statement

The authors declare that the research was conducted in the absence of any commercial or financial relationships that could be construed as a potential conflict of interest.

## References

[B1] An X. K. Hou M. L. Liu Y. D. (2016). Reference gene selection and evaluation for gene expression studies using qRT-PCR in the white-backed planthopper, *Sogatella furcifera* (Hemiptera: Delphacidae). J. Econ. Entomol. 109, 879–886. 10.1093/jee/tov333 26612891

[B2] Andersen C. L. Jensen J. L. Ørntoft T. F. (2004). Normalization of real-time quantitative reverse transcription-PCR data: a model-based variance estimation approach to identify genes suited for normalization, applied to bladder and colon cancer data sets. Cancer Res. 64, 5245–5250. 10.1158/0008-5472.CAN-04-0496 15289330

[B3] Arun A. Baumlé V. Amelot G. Nieberding C. M. (2015). Selection and validation of reference genes for qRT-PCR expression analysis of candidate genes involved in olfactory communication in the butterfly *Bicyclus anynana*. PLoS ONE 10:e0120401. 10.1371/journal.pone.0120401 25793735 PMC4368739

[B4] Arya S. K. Jain G. Upadhyay S. K. Singh H. Dixit S. Verma P. C. (2017). Reference genes validation in *Phenacoccus solenopsis* under various biotic and abiotic stress conditions. Sci. Rep. 7:13520. 10.1038/s41598-017-13925-9 29051594 PMC5648885

[B5] Bagnall N. H. Kotze A. C. (2010). Evaluation of reference genes for real-time PCR quantification of gene expression in the Australian sheep blowfly, *Lucilia cuprina*. Med. Vet. Entomol. 24, 176–181. 10.1111/j.1365-2915.2010.00866.x 20604863

[B6] Bansal R. Mamidala P. Mian M. A. Mittapalli O. Michel A. P. (2012). Validation of reference genes for gene expression studies in *Aphis glycines* (Hemiptera: Aphididae). J. Econ. Entomol. 105, 1432–1438. 10.1603/EC12095 22928326 PMC7110211

[B7] Bansal R. Mittapelly P. Chen Y. Mamidala P. Zhao C. Michel A. (2016). Quantitative RT-PCR gene evaluation and RNA interference in the brown marmorated stink bug. PLoS ONE 11:e0152730. 10.1371/journal.pone.0152730 27144586 PMC4856283

[B8] Bassan M. M. Angelotti-Mendonça J. Alves G. R. Yamamoto P. T. Mourão Filho F. D. A. A. (2017). Selection of reference genes for expression studies in *Diaphorina citri* (Hemiptera: Liviidae). J. Econ. Entomol. 110, 2623–2629. 10.1093/jee/tox253 29029285

[B9] Baumann A. Lehmann R. Beckert A. Vilcinskas A. Franta Z. (2015). Selection and evaluation of tissue specific reference genes in *Lucilia sericata* during an immune challenge. PLoS ONE 10:e0135093. 10.1371/journal.pone.0135093 26252388 PMC4529112

[B10] Bustin S. A. Benes V. Garson J. Hellemans J. Huggett J. Kubista M.. (2013). The need for transparency and good practices in the qPCR literature. Nat. Methods 10, 1063–1067. 10.1038/nmeth.2697 24173381

[B11] Bustin S. A. Benes V. Nolan T. Pfaffl M. W. (2005). Quantitative real-time RT-PCR–a perspective. J. Mol. Endocrinol. 34, 597–601. 10.1677/jme.1.01755 15956331

[B12] Cameron R. C. Duncan E. J. Dearden P. K. (2013). Stable reference genes for the measurement of transcript abundance during larval caste development in the honeybee. Apidologie 44, 357–366. 10.1007/s13592-012-0187-0

[B13] Chandra G. S. Asokan R. Manamohan M. Kumar N. K. Sita T. (2014). Evaluation of reference genes for quantitative real-time PCR normalization in cotton bollworm, *Helicoverna armigera*. Mol. Biol. 48, 813–822. 10.1134/S0026893314060156 25845233

[B14] Chang Y. W. Chen J. Y. Lu M. X. Gao Y. Tian Z. H. Gong W. R.. (2017). Selection and validation of reference genes for quantitative real-time PCR analysis under different experimental conditions in the leafminer *Liriomyza trifolii* (Diptera: Agromyzidae). PLoS ONE 12:e0181862. 10.1371/journal.pone.0181862 28746411 PMC5528903

[B15] Chapuis M. P. Tohidiesfahani D. Dodgson T. Blondin L. Ponton F. Cullen D.. (2011). Assessment and validation of a suite of reverse transcription-quantitative PCR reference genes for analyses of density-dependent behavioural plasticity in the Australian plague locust. BMC Mol. Biol. 12, 1–11. 10.1186/1471-2199-12-7 21324174 PMC3048552

[B16] Cheng D. Zhang Z. He X. Liang G. (2013). Validation of reference genes in *Solenopsis invicta* in different developmental stages, castes and tissues. PLoS ONE 8:e57718. 10.1371/journal.pone.0057718 23469057 PMC3585193

[B17] Collins C. Patel M. V. Colvin J. Bailey D. Seal S. Wolfner M. (2014). Identification and evaluation of suitable reference genes for gene expression studies in the whitefly *Bemisia tabaci* (Asia I) by reverse transcription quantitative real time PCR. J. Insect Sci. 14:63 10.1673/031.014.63 25373210 PMC4207516

[B18] Cristiano G. Grossi G. Scala A. Fanti P. Zhou J. J. Bufo S. A. (2016). Validation of reference genes for qRT-PCR analysis in *Megoura viciae* (Hemiptera Aphididae). B. Insectol. 69, 229–238.

[B19] Dai T. M. Lü Z. C. Liu W. X. Wan F. H. (2017). Selection and validation of reference genes for qRT-PCR analysis during biological invasions: the thermal adaptability of *Bemisia tabaci* MED. PLoS ONE 12:e0173821. 10.1371/journal.pone.0173821 28323834 PMC5360248

[B20] Dzaki N. Ramli K. N. Azlan A. Ishak I. H. Azzam G. (2017). Evaluation of reference genes at different developmental stages for quantitative real-time PCR in *Aedes aegypti*. Sci. Rep. 7:43618. 10.1038/srep43618 28300076 PMC5353741

[B21] Fu W. Xie W. Zhang Z. Wang S. Wu Q. Liu Y.. (2013). Exploring valid reference genes for quantitative real-time PCR analysis in *Plutella xylostella* (Lepidoptera: Plutellidae). Int. J. Biol. Sci. 9:792. 10.7150/ijbs.5862 23983612 PMC3753443

[B22] Galetto L. Bosco D. Marzach,ì C. (2013). Selection of reference genes from two leafhopper species challenged by phytoplasma infection, for gene expression studies by RT-qPCR. BMC Res. Notes 6:409. 10.1186/1756-0500-6-409 24119747 PMC3852609

[B23] Gao X. K. Zhang S. Luo J. Y. Wang C. Y. Lü L. M. Zhang L. J.. (2017). Comprehensive evaluation of candidate reference genes for gene expression studies in *Lysiphlebia japonica* (Hymenoptera: Aphidiidae) using RT-qPCR. Gene 637, 211–218 10.1016/j.gene.2017.09.057 28964897

[B24] Guo H. Jiang L. Xia Q. (2016). Selection of reference genes for analysis of stress-responsive genes after challenge with viruses and temperature changes in the silkworm *Bombyx mori*. Mol. Genet. Genomics 291:999. 10.1007/s00438-015-1125-4 26437927

[B25] Hornáková D. Matousková P. Kindl J. Valterová I. Pichová I. (2010). Selection of reference genes for real-time polymerase chain reaction analysis in tissues from *Bombus terrestris* and *Bombus lucorum* of different ages. Anal. Biochem. 397, 118–120. 10.1016/j.ab.2009.09.019 19751695

[B26] Ibanez F. Tamborindeguy C. (2016). Selection of reference genes for expression analysis in the potato psyllid, *Bactericera cockerelli*. Insect Mol. Biol. 25, 227–238. 10.1111/imb.12219 26936438

[B27] Jiang H. B. Liu Y. H. Tang P. A. Zhou A. W. Wang J. J. (2010). Validation of endogenous reference genes for insecticide-induced and developmental expression profiling of *Liposcelis bostsrychophila* (Psocoptera: Liposcelididae). Mol. Biol. Rep. 37:1019. 10.1007/s11033-009-9803-0 19757170

[B28] Kalushkov P. Hodek I. (2004). The effects of thirteen species of aphids on some life history parameters of the ladybird *Coccinella septempunctata*. Biol. Control 49, 21–32. 10.1023/B:BICO.0000009385.90333.b4

[B29] Kang Z. W. Liu F. H. Tian H. G. Zhang M. Guo S. S. Liu T. X. (2017). Evaluation of the reference genes for expression analysis using quantitative real-time polymerase chain reaction in the green peach aphid, *Myzus persicae*. Insect Sci. 24, 222–234. 10.1111/1744-7917.12310 26749166

[B30] Koramutla M. K. Aminedi R. Bhattacharya R. (2016). Comprehensive evaluation of candidate reference genes for qRT-PCR studies of gene expression in mustard aphid, *Lipaphis erysimi* (Kalt). Sci. Rep. 6:25883. 10.1038/srep25883 27165720 PMC4863174

[B31] Li R. Xie W. Wang S. Wu Q. Yang N. Yang X.. (2013). Reference gene selection for qRT-PCR analysis in the sweetpotato whitefly, *Bemisia tabaci* (Hemiptera: Aleyrodidae). PLoS ONE 8:e53006. 10.1371/journal.pone.0053006 23308130 PMC3540095

[B32] Liang P. Guo Y. Zhou X. Gao X. (2014). Expression profiling in *Bemisia tabaci* under insecticide treatment: indicating the necessity for custom reference gene selection. PLoS ONE 9:e87514. 10.1371/journal.pone.0087514 24498122 PMC3909111

[B33] Ling D. Salvaterra P. M. (2011). Robust RT-qPCR data normalization: validation and selection of internal reference genes during post-experimental data analysis. PLoS ONE 6:e17762. 10.1371/journal.pone.0017762 21423626 PMC3058000

[B34] Liu G. Qiu X. Cao L. Zhang Y. Zhan Z. Han R. (2016). Evaluation of reference genes for reverse transcription quantitative PCR studies of physiological responses in the ghost moth, *Thitarodes armoricanus* (Lepidoptera, Hepialidae). PLoS ONE 11:e0159060. 10.1371/journal.pone.0159060 27392023 PMC4938418

[B35] Lord J. C. Hartzer K. Toutges M. Oppert B. (2010). Evaluation of quantitative PCR reference genes for gene expression studies in *Tribolium castaneum* after fungal challenge. J. Microbiol. Meth. 80, 219–221. 10.1016/j.mimet.2009.12.007 20026205

[B36] Lu Y. Yuan M. Gao X. Kang T. Zhan S. Wan H.. (2013). Identification and validation of reference genes for gene expression analysis using quantitative PCR in *Spodoptera litura* (Lepidoptera: Noctuidae). PLoS ONE 8:e68059. 10.1371/journal.pone.0068059 23874494 PMC3706614

[B37] Lü Z. C. Wang L. H. Dai R. L. Zhang G. F. Guo J. Y. Wan F. H. (2014). Evaluation of endogenous reference genes of *Bactrocera (tetradacus) minax* by gene expression profiling under various experimental conditions. Fla. Entomol. 97, 597–604. 10.1653/024.097.0235

[B38] Lü Z. H. Pan H. P. Zhang W. Ding T. B. Chu D. (2017). Reference gene selection for RT-qPCR analysis in two invasive whiteflies after the acquisition of vectored or non-vectored viruses. J. Asia-Pac. Entomol. 21, 19–24. 10.1016/j.aspen.2017.10.001

[B39] Ma K. S. Li F. Liang P. Z. Chen X. W. Liu Y. Gao X. W. (2016). Identification and validation of reference genes for the normalization of gene expression data in qRT-PCR analysis in *Aphis gossypii* (Hemiptera: Aphididae). J. Insect Sci. 16:17. 10.1093/jisesa/iew003 28076279 PMC5778981

[B40] Majerowicz D. Alves-Bezerra M. Logullo R. Fonseca-de-Souza A. L. Meyer-Fernandes J. R. Braz G. R. C.. (2011). Looking for reference genes for real-time quantitative PCR experiments in *Rhodnius prolixus*, (Hemiptera: Reduviidae). Insect Mol. Biol. 20, 713–722. 10.1111/j.1365-2583.2011.01101.x 21929722

[B41] Mamidala P. Rajarapu S. P. Jones S. C. Mittapalli O. (2011). Identification and validation of reference genes for quantitative real-time polymerase chain reaction in *Cimex lectularius*. J. Med. Entomol. 48, 947–951. 10.1603/ME10262 21845960

[B42] Marchal E. Hult E. F. Huang J. Tobe S. S. (2013). Sequencing and validation of housekeeping genes for quantitative real-time PCR during the gonadotrophic cycle of *Diploptera punctata*. BMC Res. Notes 6:237. 10.1186/1756-0500-6-237 23777660 PMC3750588

[B43] Maroniche G. A. Sagadín M. Mongelli V. C. Truol G. A. Del V. M. (2011). Reference gene selection for gene expression studies using RT-qPCR in virus-infected planthoppers. Virol. J. 8:308. 10.1186/1743-422X-8-308 21679431 PMC3142240

[B44] Matta B. P. Bitner-Mathé B. C. Alves-Ferreira M. (2011). Getting real with real-time qPCR: a case study of reference gene selection for morphological variation in *Drosophila melanogaster*, wings. Dev. Genes Evol. 221, 49–57. 10.1007/s00427-011-0356-6 21509536

[B45] Mcintosh C. H. Baird J. Zinser E. Woods D. J. Campbell E. M. Bowman A. S. (2016). Reference gene selection and RNA preservation protocol in the cat flea, *Ctenocephalides felis*, for gene expression studies. Parasitology 143, 1532–1542. 10.1017/S0031182016001025 27406059

[B46] Nakamura A. M. Chahadehlers S. Lima A. L. Taniguti C. H. Sobrinho I. J. Jr. Torres F. R.. (2016). Reference genes for accessing differential expression among developmental stages and analysis of differential expression of OBP genes in *Anastrepha obliqua*. Sci. Rep. 6:17480. 10.1038/srep17480 26818909 PMC4730201

[B47] Niu J. Cappelle K. de Miranda J. R. Smagghe G. Meeus I. (2014). Analysis of reference gene stability after Israeli acute paralysis virus infection in bumblebees *Bombus terrestris*. J. Invertebr. Pathol. 115:76. 10.1016/j.jip.2013.10.011 24184950

[B48] Omondi B. A. Latorre-Estivalis J. M. Oliveira I. H. Ignell R. Lorenzo M. G. (2015). Evaluation of reference genes for insect olfaction studies. Parasite. Vector. 8:243. 10.1186/s13071-015-0862-x 25896676 PMC4417234

[B49] Paim R. M. Pereira M. H. Di Ponzio R. Rodrigues J. O. Guarneri A. A. Gontijo N. F.. (2012). Validation of reference genes for expression analysis in the salivary gland and the intestine of *Rhodnius prolixus* (Hemiptera, Reduviidae) under different experimental conditions by quantitative real-time PCR. BMC Res. Notes 5:128. 10.1186/1756-0500-5-128 22395020 PMC3337225

[B50] Pan H. Yang X. Bidne K. Hellmich R. L. Siegfried B. D. Zhou X. (2015a). Selection of reference genes for RT-qPCR analysis in the monarch butterfly, *Danaus plexippus* (L.), a migrating bio-indicator. PLoS ONE 10:e0129482. 10.1371/journal.pone.0129482 26030778 PMC4452232

[B51] Pan H. Yang X. Siegfried B. D. Zhou X. (2015b). A comprehensive selection of reference genes for RT-qPCR analysis in a predatory lady beetle, *Hippodamia convergens* (Coleoptera: Coccinellidae). PLoS ONE 10:e0125868. 10.1371/journal.pone.0125868 25915640 PMC4411045

[B52] Pfaffl M. W. Tichopad A. Prgomet C. Neuvians T. P. (2004). Determination of stable housekeeping genes, differentially regulated target genes and sample integrity: BestKeeper—excel-based tool using pair-wise correlations. Biotechnol. Lett. 26, 509–515. 10.1023/B:BILE.0000019559.84305.47 15127793

[B53] Piron Prunier F. Chouteau M. Whibley A. Joron M. Llaurens V. (2016). Selection of valid reference genes for reverse transcription quantitative PCR analysis in *Heliconius numata* (Lepidoptera: Nymphalidae). J. Insect Sci. 16:50. 10.1093/jisesa/iew034 27271971 PMC4896466

[B54] Ponton F. Chapuis M. P. Pernice M. Sword G. A. Simpson S. J. (2011). Evaluation of potential reference genes for reverse transcription-qPCR studies of physiological responses in *Drosophila melanogaster*. J. Insect Physiol. 57, 840–850. 10.1016/j.jinsphys.2011.03.014 21435341

[B55] Rajarapu S. P. Mamidala P. Mittapalli O. (2012). Validation of reference genes for gene expression studies in the emerald ash borer (*Agrilus planipennis*). Insect Sci. 19, 41–46. 10.1111/j.1744-7917.2011.01447.x

[B56] Reim T. Thamm M. Rolke D. Blenau W. Scheiner R. (2013). Suitability of three common reference genes for quantitative real-time PCR in honey bees. Apidologie 44, 342–350. 10.1007/s13592-012-0184-3

[B57] Ridgeway J. A. Timm A. E. (2015). Reference gene selection for quantitative real-time PCR normalization in larvae of three species of Grapholitini (Lepidoptera: Tortricidae). PLoS ONE 10:e0129026. 10.1371/journal.pone.0129026 26030743 PMC4450875

[B58] Rodrigues T. B. Dhandapani R. K. Duan J. J. Palli S. R. (2017). RNA interference in the Asian longhorned beetle: identification of key RNAi genes and reference genes for RT-qPCR. Sci. Rep. 7:8913. 10.1038/s41598-017-08813-1 28827780 PMC5566337

[B59] Rodrigues T. B. Khajuria C. Wang H. Matz N. Cunha C. D. Valicente F. H.. (2013). Validation of reference housekeeping genes for gene expression studies in western corn rootworm (*Diabrotica virgifera virgifera*). PLoS ONE 9:e109825. 10.1371/journal.pone.0109825 25356627 PMC4214676

[B60] Sagri E. Koskinioti P. Gregoriou M. E. Tsoumani K. T. Bassiakos Y. C. Mathiopoulos K. D. (2017). Housekeeping in Tephritid insects: the best gene choice for expression analyses in the medfly and the olive fly. Sci. Rep. 7:45634. 10.1038/srep45634 28368031 PMC5377319

[B61] Sang J. Wang Z. Li M. Cao J. Niu G. Xia L.. (2017). ICG: a wiki-driven knowledgebase of internal control genes for RT-qPCR normalization. Nucleic Acids Res. 46, D121–D126. 10.1093/nar/gkx875 29036693 PMC5753184

[B62] Sang W. He L. Wang X. P. Zhusalzman K. Lei C. L. (2015). Evaluation of reference genes for RT-qPCR in *Tribolium castaneum* (Coleoptera: Tenebrionidae) under UVB stress. Environ. Entomol. 44:418. 10.1093/ee/nvv010 26313197

[B63] Scharlaken B. Graaf D. C. D. Goossens K. Brunain M. Peelman L. J. Jacobs F. J. (2008). Reference gene selection for insect expression studies using quantitative real-time PCR: the head of the honeybee, *Apis mellifera*, after a bacterial challenge. J. Insect Sci. 8, 1–10. 10.1673/031.008.3301

[B64] Shakeel M. Zhu X. Kang T. Wan H. Li J. (2015). Selection and evaluation of reference genes for quantitative gene expression studies in cotton bollworm, *Helicoverpa armigera*, (Lepidoptera: Noctuidae). J. Asia-Pac. Entomol. 18, 123–130. 10.1016/j.aspen.2015.01.001

[B65] Shang F. Wei D. D. Jiang X. Z. Wei D. Shen G. M. Feng Y. C.. (2015). Reference gene validation for quantitative PCR under various biotic and abiotic stress conditions in *Toxoptera citricida* (Hemiptera, Aphidiae). J. Econ. Entomol. 108, 2040–2047. 10.1093/jee/tov184 26470351

[B66] Shen G. M. Huang Y. Jiang X. Z. Dou W. Wang J. J. (2013). Effect of β-cypermethrin exposure on the stability of nine housekeeping genes in *Bactrocera dorsalis* (Diptera: Tephritidae). Fla. Entomol. 96, 442–450. 10.1653/024.096.0208

[B67] Shen G. M. Jiang H. B. Wang X. N. Wang J. J. (2010). Evaluation of endogenous references for gene expression profiling in different tissues of the oriental fruit fly *Bactrocera dorsalis* (Diptera: Tephritidae). BMC Mol. Biol. 11:76. 10.1186/1471-2199-11-76 20923571 PMC2972281

[B68] Shi C. Yang F. Zhu X. Du E. Yang Y. Wang S.. (2016). Evaluation of housekeeping genes for quantitative real-time PCR analysis of *Bradysia odoriphaga* (Diptera: Sciaridae). Int. J. Mol. Sci. 17:1034. 10.3390/ijms17071034 27399679 PMC4964410

[B69] Shi X. Q. Guo W. C. Wan P. J. Zhou L. T. Ren X. L. Ahmat T.. (2013). Validation of reference genes for expression analysis by quantitative real-time PCR in *Leptinotarsa decemlineata* (say). BMC Res. Notes 6:93. 10.1186/1756-0500-6-93 23497596 PMC3600673

[B70] Sinha D. K. Smith C. M. (2014). Selection of reference genes for expression analysis in *Diuraphis noxia* (Hemiptera: Aphididae) fed on resistant and susceptible wheat plants. Sci. Rep. 4:5059. 10.1038/srep05059 24862828 PMC4034006

[B71] Strube C. Buschbaum S. Wolken S. Schnieder T. (2008). Evaluation of reference genes for quantitative real-time PCR to investigate protein disulfide isomerase transcription pattern in the bovine lungworm *Dictyocaulus viviparus*. Gene 425, 36–43. 10.1016/j.gene.2008.08.001 18761062

[B72] Su Y. L. He W. B. Wang J. Li J. M. Liu S. S. Wang X. W. (2013). Selection of endogenous reference genes for gene expression analysis in the Mediterranean species of the *Bemisia tabaci* (Hemiptera: Aleyrodidae) complex. J. Econ. Entomol. 106, 1446–1455. 10.1603/EC12459 23865213

[B73] Sun M. Lu M. X. Tang X. T. Du Y. Z. (2015). Exploring valid reference genes for quantitative real-time PCR analysis in *Sesamia inferens* (Lepidoptera: Noctuidae). PLoS ONE 10:e0115979. 10.1371/journal.pone.0115979 25585250 PMC4293147

[B74] Tan Q. Q. Zhu L. Li Y. Liu W. Ma W. H. Lei C. L.. (2015). A *de novo* transcriptome and valid reference genes for quantitative real-time PCR in *Colaphellus bowringi*. PLoS ONE 10:e0118693. 10.1371/journal.pone.0118693 25692689 PMC4334893

[B75] Tan Y. Zhou X. R. Pang B. P. (2017). Reference gene selection and evaluation for expression analysis using qRT-PCR in *Galeruca daurica* (Joannis). B. Entomol. Res. 107, 359–368. 10.1017/S0007485316000948 27819206

[B76] Tang P. A. Duan J. Y. Wu H. J. Ju X. R. Yuan M. L. (2017). Reference gene selection to determine differences in mitochondrial gene expressions in phosphine-susceptible and phosphine-resistant strains of *Cryptolestes ferrugineus*, using qRT-PCR. Sci. Rep. 7:7047. 10.1038/s41598-017-07430-2 28765619 PMC5539111

[B77] Teng X. Zhang Z. He G. Yang L. Li F. (2012). Validation of reference genes for quantitative expression analysis by real-time RT-PCR in four lepidopteran insects. J. Insect Sci. 12, 1–17. 10.1673/031.012.6001 22938136 PMC3481461

[B78] Toutges M. J. Hartzer K. Lord J. Oppert B. (2010). Evaluation of reference genes for quantitative polymerase chain reaction across life cycle stages and tissue types of *Tribolium castaneum*. J. Agr. Food Chem. 58:8948. 10.1021/jf101603j 20672839

[B79] Van Hiel M. B. Van Wielendaele P Temmerman L. Van S. S. Vuerinckx K. Huybrechts R.. (2009). Identification and validation of housekeeping genes in brains of the desert locust *Schistocerca gregaria* under different developmental conditions. BMC Mol. Biol. 10:56. 10.1186/1471-2199-10-56 19508726 PMC2700112

[B80] Vandesompele J. De Preter K. Pattyn F. Poppe B. Van Roy N. De Paepe A.. (2002). Accurate normalization of real-time quantitative RT-PCR data by geometric averaging of multiple internal control genes. Genome Biol. 3:research0034-1. 10.1186/gb-2002-3-7-research0034 12184808 PMC126239

[B81] Wang W. X. Lai F. X. Li K. L. Fu Q. (2014). Selection of reference genes for gene expression analysis in *Nilaparvata lugens* with different levels of virulence on rice by quantitative real-time PCR. Rice Sci. 21, 305–311. 10.1016/S1672-6308(14)60272-9

[B82] Wang X. Y. Xiong M. Wang J. L. Lei C. L. Zhu F. (2015). Reference gene stability of a synanthropic fly, *Chrysomya megacephala*. Parasit. Vectors 8:565. 10.1186/s13071-015-1175-9 26515169 PMC4625446

[B83] Wang Y. Wang Z. K. Huang Y. Liao Y. F. Yin Y. P. (2014). Identification of suitable reference genes for gene expression studies by qRT-PCR in the blister beetle *Mylabris cichorii*. J. Insect Sci. 14:94. 10.1093/jis/14.1.94 25368050 PMC4212844

[B84] Wu K. Liu W. Mar T. Liu Y. Wu Y. Wang X. (2014). Sequencing and validation of reference genes to analyze endogenous gene expression and quantify yellow dwarf viruses using RT-qPCR in viruliferous *Rhopalosiphum padi*. PLoS ONE 9:e97038. 10.1371/journal.pone.0097038 24810421 PMC4014588

[B85] Xu J. Lu M. X. Cui Y. D. Du Y. Z. (2017). Selection and evaluation of reference genes for expression analysis using qRT-PCR in *Chilo suppressalis* (Lepidoptera: Pyralidae). J. Econ. Entomol. 110, 683–691. 10.1093/jee/tow297 28115499

[B86] Yang C. Li H. Pan H. Ma Y. Zhang D. Liu Y.. (2015a). Stable reference gene selection for RT-qPCR analysis in nonviruliferous and viruliferous *Frankliniella occidentalis*. PLoS ONE 10:e0135207. 10.1371/journal.pone.0135207 26244556 PMC4526564

[B87] Yang C. Pan H. Liu Y. Zhou X. (2014). Selection of reference genes for expression analysis using quantitative real-time PCR in the pea aphid, *Acyrthosiphon pisum* (Harris) (Hemiptera, Aphidiae). PLoS ONE 9:e110454. 10.1371/journal.pone.0110454 25423476 PMC4244036

[B88] Yang C. Pan H. Liu Y. Zhou X. (2015b). Temperature and development impacts on housekeeping gene expression in cowpea aphid, *Aphis craccivora* (Hemiptera: Aphidiae). PLoS ONE 10:e0130593. 10.1371/journal.pone.0130593 26090683 PMC4474611

[B89] Yang C. Pan H. Noland J. E. Zhang D. Zhang Z. Liu Y.. (2015c). Selection of reference genes for RT-qPCR analysis in a predatory biological control agent, *Coleomegilla maculata* (Coleoptera: Coccinellidae). Sci. Rep. 5:18201. 10.1038/srep18201 26656102 PMC4674751

[B90] Yang C. Preisser E. L. Zhang H. Liu Y. Dai L. Pan H. (2016). Selection of reference genes for RT-qPCR analysis in *Coccinella septempunctata* to assess un-intended effects of RNAi transgenic plants. Front. Plant Sci. 7:e53006 10.3389/fpls.2016.01672 PMC5099537 27877186

[B91] Yang Q. Li Z. Cao J. Zhang S. Zhang H. Wu X.. (2014). Selection and assessment of reference genes for quantitative PCR normalization in migratory locust, *Locusta migratoria*, (Orthoptera: Acrididae). PLoS ONE 9:e98164. 10.1371/journal.pone.0098164 24887329 PMC4041718

[B92] Yang X. Pan H. Yuan L. Zhou X. (2018). Reference gene selection for RT-qPCR analysis in *Harmonia axyridis*, a global invasive lady beetle. Sci. Rep. 8:2689. 10.1038/s41598-018-20612-w 29426915 PMC5807316

[B93] Yu S. H. Pu Y. Sun T. Qi Q. Wang X. Q. Xu D. L.. (2016). Identification and evaluation of reference genes in the Chinese white wax scale insect *Ericerus pela*. Springerplus 5, 1–8. 10.1186/s40064-016-2548-z 27390632 PMC4916112

[B94] Yuan M. Lu Y. Zhu X. Wan H. Shakeel M. Zhan S.. (2014). Selection and evaluation of potential reference genes for gene expression analysis in the brown planthopper, *Nilaparvata lugens* (Hemiptera: Delphacidae) using reverse-transcription quantitative PCR. PLoS ONE 9:e86503. 10.1371/journal.pone.0086503 24466124 PMC3900570

[B95] Zhai Y. Lin Q. Zhou X. Zhang X. Liu T. Yu Y. (2014). Identification and validation of reference genes for quantitative real-time PCR in *Drosophila suzukii* (Diptera: Drosophilidae). PLoS ONE 9:e106800. 10.1371/journal.pone.0106800 25198611 PMC4157791

[B96] Zhang L. Zhang Q. L. Wang X. T. Yang X. Z. Li X. P. Yuan M. L. (2017). Selection of reference genes for qRT-PCR and expression analysis of high-altitude-related genes in grassland caterpillars (Lepidoptera: Erebidae: *Gynaephora*) along an altitude gradient. Ecol. Evol. 7, 9054–9065. 10.1002/ece3.3431 29152197 PMC5677504

[B97] Zhang S. An S. Li Z. Wu F. Yang Q. Liu Y.. (2015). Identification and validation of reference genes for normalization of gene expression analysis using qRT-PCR in *Helicoverpa armigera* (Lepidoptera: Noctuidae). Gene 555, 393–402. 10.1016/j.gene.2014.11.038 25447918

[B98] Zheng Y. T. Li H. B. Lu M. X. Du Y. Z. (2014). Evaluation and validation of reference genes for qRT-PCR normalization in *Frankliniella occidentalis* (Thysanoptera: Thripidae). PLoS ONE 9:e111369. 10.1371/journal.pone.0111369 25356721 PMC4214748

[B99] Zhong M. Wang X. Wen J. Cai J. Wu C. Aly S. M. (2013). Selection of reference genes for quantitative gene expression studies in the house fly (*Musca domestica* L.) using reverse transcription quantitative real-time PCR. Acta Bioch. Bioph. Sin. 45:1069. 10.1093/abbs/gmt111 24113091

[B100] Zhu X. Yuan M. Shakeel M. Zhang Y. Wang S. Wang X.. (2014). Selection and evaluation of reference genes for expression analysis using qRT-PCR in the beet armyworm *Spodoptera exigua* (Hübner) (Lepidoptera: Noctuidae). PLoS ONE 9:e84730. 10.1371/journal.pone.0084730 24454743 PMC3893131

[B101] Zotti M. Dos Santos E. A. Cagliari D. Christiaens O. Taning C. N. T. Smagghe G. (2018). RNA interference technology in crop protection against arthropod pests, pathogens and nematodes. Pest Manag. Sci. 74, 1239–1250. 10.1002/ps.4813 29194942

